# Genetic Diversity in Normal Cell Populations is the Earliest Stage of Oncogenesis Leading to Intra-Tumor Heterogeneity

**DOI:** 10.3389/fonc.2013.00061

**Published:** 2013-04-05

**Authors:** Cory L. Howk, Zachary Voller, Brandon B. Beck, Donghai Dai

**Affiliations:** ^1^Department of Obstetrics and Gynecology, University of IowaIowa City, IA, USA; ^2^Department of Mathematics, Iowa State UniversityAmes, IA, USA

**Keywords:** evolution, oncogenesis, genetic mutation, endometrial cancer, fitness, phylogenetic analysis, tumor heterogeneity, mathematical modeling

## Abstract

Random mutations and epigenetic alterations provide a rich substrate for microevolutionary phenomena to occur in proliferating epithelial tissues. Genetic diversity resulting from random mutations in normal cells is critically important for understanding the genetic basis of oncogenesis. However, evaluation of the cell-specific role of individual (epi-)genetic alterations in living tissues is extremely difficult from a direct experimental perspective. For this purpose, we have developed a single cell model to describe the fate of every cell in the uterine epithelium and to simulate occurrence of the first cancer cell. Computational simulations have shown that a baseline mutation rate of two mutations per cell division is sufficient to explain sporadic endometrial cancer as a rare evolutionary consequence with an incidence similar to that reported in SEER data. Simulation of the entire oncogenic process has allowed us to analyze the features of the tumor-initiating cells and their clonal expansion. Analysis of the malignant features of individual cancer cells, such as de-differentiation status, proliferation potential, and immortalization status, permits a mathematical characterization of malignancy at the single cell level and a comparison of intra-tumor heterogeneity between individual tumors. We found, under the conditions specified, that cancer stem cells account for approximately 7% of the total cancer cell population. Therefore, our mathematical modeling describes the genetic diversity and evolution in a normal cell population at the early stages of oncogenesis and characterizes intra-tumor heterogeneity. This model has explored the role of accumulation of a large number of genetic alterations in oncogenesis as an alternative to traditional biological approaches emphasizing the driving role of a small number of genetic mutations. A quantitative description of the contribution of a large set of genetic alterations will allow the investigation of the impact of environmental factors on the growth advantage of and selection pressure on individual cancer cells for tumor progression.

## Introduction

An evolutionary model has been established to describe the entire process of tumor development in colorectal cancer with detailed molecular mechanisms for the stepwise oncogenic progression driven by sequential accumulation of several genetic mutations (Fearon and Vogelstein, 1990; Jones et al., 2008a). However, in our view, this model can be expanded to understand evolution among a population of normal cells in the uterine epithelium with inclusion of random mutations. Several studies have estimated the mutation rates in normal cells to be around 10^−7^ per cell per generation (for a specific gene) through measurement of the frequency of mutations in the gene in proliferating cells (Elmore et al., 1983; Araten et al., 2005). The more accurate estimates are done in a living tissue and a rate of ≈5–10 × 10^−10^ mutations per base pair per cell per generation is reported (Jones et al., 2008a). This rate can be approximately translated into about two to three mutations per cell per division. This reported mutation rate of two to three random mutations per cell per generation would produce billions of mutations in the proliferating uterine epithelial tissue and may be sufficient to explain the large number of genetic mutations uncovered in human tumors (Gallo et al., 2012; Kuhn et al., 2012; Liang et al., 2012). Interestingly, these studies have not found a significant difference in the mutation rate between normal and transformed cells (Elmore et al., 1983; Araten et al., 2005; Jones et al., 2008a), indicating that the genetic diversity universally reported in cancer cell populations may be present in normal cell populations as well, serving as fertile ground for evolution at the earliest stage of oncogenesis. Therefore, genetic mutations in normal cells can provide significant genetic diversity for subsequent selection, allowing for a unique, albeit extremely rare, consequence: a cell may escape the typical fate of normal cells and become immortalized.

However, the process of evolution in a normal cell population is rarely a popular cancer research subject. Normal cells in a tissue are often not considered to harbor any dysfunctional mutations nor are they considered to demonstrate any phenotype commonly seen in cancer. Furthermore, any suggestion that minor random mutations are sufficient for oncogenesis in some cancers may be seen as a contradiction to the genetic theory that certain notable genetic mutations and oncogenic pathways are the driving forces for tumor development. These seeming contradictions can be reconciled by considering that a significantly larger number of pathways than was commonly believed are present in well-developed tumors (Jones et al., 2008b), meaning that the genetic slot machine for transformation of an individual cell has many reels. Phenotypically normal cells, with no apparent growth advantage, may quietly harbor multiple accumulated alterations in multiple pathways before transformation by a single major mutation or by minor mutations in remaining key pathways. While the chance of complete transformation of an individual cell may be negligible, genetic diversity represents the non-negligible collective chances of many individual cells, each with a particular set of mutations after a number of generations with a steady mutation rate.

The appearance of the first cancer cell, the tumor-initiating cancer cell (TICC) which propagates to form the entire cancer cell population in a tumor, seems to be an extremely rare occurrence. For instance, endometrial cancer incidence is about 6 per 100,000 women at reproductive age according to the SEER database (2008, female, all races, <50 years) and the peak cell number in the uterine epithelium is several billion with monthly turnover, which gives an approximate probability of the occurrence of the TICC of less than 5 × 10^−15^ per normal cell per year. This manuscript, utilizing mathematical modeling and numerical simulation, tests whether the baseline mutation rate in a normal cell population, such as the uterine epithelium, is sufficient for the rare occurrence of a TICC. Simulation of the longitudinal and prospective process of tumor initiation and development, including following the evolution of individual normal cell lines in the uterine epithelium, has allowed us to describe the clonal progression of a TICC into a clinically detectable tumor.

## Materials and Methods

The goal of this manuscript is to explore whether the baseline mutation rate in a normal endometrial cell population is sufficient to explain endometrial cancer incidence. We will also explore whether description of the fate of every single cell in our model can demonstrate in sufficient detail the development of heterogeneity within the mass, and the corresponding properties of the ancestor cells of endometrial tumors. This is analyzed through numerical simulations of a recently published model for the proliferation of uterine epithelial cells (Dai et al., 2011).

### Outline of cell propagation

The mathematical model under consideration views the proliferation of epithelial cells in terms of a continuous-time bifurcating process. The simulation begins with an initial progenitor cell. The time required for the cell to either divide or die is governed by a set of equations describing various properties of the cell (Eqs 1–7, individual variables are described in Tables 1 and 2). In the event of division, the daughter cells inherit their properties from the parent cell, with the quantitative values of the properties subject to stochastic variation. We then follow the fates of each daughter cell, which follow Eqs 1–7 independently. The cells are simultaneously viewed as traversing a differentiation pathway, with each cell existing along a spectrum from progenitor cell to a fully differentiated descendant clone typically seen in the uterine epithelium (Dai et al., 2011). Therefore the cell’s properties are also influenced due to this “biological progression.” The size of the uterine epithelium is determined by the total number of descendant cells existing at time *t*. The fate of each individual constituent cell is calculated through Monte Carlo simulation.

(1) $$\begin{array}{llll} & \text{Cell}\;\text{cycle status value: } & & \\ & \;c\left(t\right)=\int_{t_{n}}^{t}\alpha \left(s\right)ds\text{, where }t_{n}\;\text{denote}\;\text{the}\;\text{cell’s}\;\text{birth}\;\text{time} & \end{array}$$

(2) $$\begin{array}{llll} & \text{Programmed proliferation potential: } & & \\ & \;\alpha_{p}\left(t\right)=\frac{1}{7}\left(10-g\left(t\right)\right)\;g\left(t\right) & \end{array}$$

(3) $$\begin{array}{llll} & \text{Programmed differentiation coefficient: } & & \\ & \;k_{p}\left(t\right)=3.78\left[1-e^{-0.4\cdot g\left(t\right)}\right]+0.03g\left(t\right) & \end{array}$$

(4) $$\begin{array}{lll} & \text{Generation number: }g\left(t\right)=1+\text{floor}\left(\int_{0}^{t}\left|\alpha \left(s\right)\right|ds\right) & \end{array}$$

(5) $$\begin{array}{lll} & \text{Resistance potential: }r\left(t\right)=k\left(t\right)\left(\alpha_{p}\left(t\right)-\alpha \left(t\right)\right) & \end{array}$$

(6) $$\begin{array}{lll} & \text{Differentiation coefficient: }k\left(t\right)=k_{p}\left(t\right)+\overset{n}{\underset{i=1}{\sum }}m_{i} & \end{array}$$

(7) $$\begin{array}{lll} & \text{Proliferation potential rate of change: }\frac{d\alpha }{dt}=r\left(t\right)+\beta \left(t\right) & \end{array}$$

**Table 1:** **Terms for hypothetical cellular growth of a single cell**.

Term	Definition	Unit	Description
*c*(*t*)	Measurement of the status of cell cycle of a cell with a numerical value between −1 and +1	Cycle	The status of a cell cycle is provided with a numerical value in order to describe the quantitative progression of cell proliferation. A cell cycle exists between two endpoints: death and birth (of two daughter cells). In either case, the cell ceases to exist. A cell divides if *c*(*t*) = 1, dies if *c*(*t*) = −1, for some *t* > *t_n_*, where *t_n_* is the time that the cell was born
*N*(*t*)	Size of a tissue or a mass at time *t*	Cell	The total number of cells in a tissue or a mass at time *t* with summation of the value of all individual cells. A clone is comprised of all descendant cells from a progenitor cell borne from asymmetrical division of a tissue stem cell
*t*	Physical time, as it relates to patient age and menstrual cycle	Month	It is the physical time and can be assigned with a unit of day, month, or year. We assume that 1 year = 12 months and 1 month = 30 days for convenience
α*_p_*(*t*)	(Programed) proliferation potential (Eq. 2)	Cycles/month	Programed rate of a cell’s multiplication according to the cell’s progression in clonal development (progression of generations) and expressed as the number of cell cycles per unit time
*k_p_*(*t*)	(Programed) differentiation coefficient (Eq. 3)	1/month	Measurement of a cell’s differentiation status, commonly with a range from 0 to *K*_max_ (a tissue specific constant)
*g*(*t*)	Generation number (Eq. 4)	Cycle	Measurement of lineage progression in a clone and cellular senescence. A daughter cell assumes a new generation value of *g* + 1 with *g* as the parent generation number. It has the same unit as the cell cycle. It represents how a cell perceives senescence, and is determined by its cellular mechanism, for instance by telomere length. Although *g*(*t*) and division (*d*) synchronize most of time, there is a possibility that they may differ. For instance, active telomerase may maintain telomere length after many divisions

*These terms are for cells living under conditions free of any genetic insults and environmental influences, a hypothetical scenario used as a frame of reference to study the effect of genetic and environmental factors on cell growth*.

**Table 2:** **Terms for the growth of a single cell**.

Term	Definition	Unit	Description
*m_i_*	Mutational coefficient (Eq. 6)	1/month	Quantifies the effect of each genetic alteration on a cell’s ability to maintain differentiation status, *k*(*t*)
α(*t*)	Proliferation potential (Eq. 7)	Cycles/month	A measurement of the number of completed cell cycle per unit time. A cell’s proliferation potential is the function of resistance potential (*r*) and environmental stimulation (β) over time (*t*) in Eq. 7, indicating the pace of cell cycling under influence. Therefore, cell death induced by anti-growth signals can be simulated by a negative α induced by a negative β over time
*k*(*t*)	Differentiation coefficient (Eq. 6)	1/month	Measurement of a cell’s differentiation status under influence as the sum of programmed differentiation coefficient and mutational effect
*r*(*t*)	Resistance potential (Eq. 6)	Cycles/month^2^	Measurement of a cell’s inherent ability to adhere to the development program by restoring α(*t*) to α*_p_*(*t*) which will lead to the control of cell number and progression of differentiation
β(*t*)	Environmental coefficient (Eq. 7)	Cycles/month^2^	All environmental factors affecting cell multiplication. Hormonal stimulation on cell proliferation is an example

*These terms are for experimental measurement of (clonal) cellular growth under our experimental observations with genetic insults and under environmental influences*.

The cell cycle status *c*(*t*) of a cell, governed by the cell’s growth rate (proliferation potential) α(*t*), denotes the progression toward apoptosis (death) or division (bifurcation) in the branching process. When a cell is born at a time *t_n_*, this value is 0. If *c*(t*) = 1 for some t* > *t_n_*, the cell undergoes division into two daughter cells, while if *c*(t*) = −1 for some t* > *t_n_*, the cell undergoes apoptosis. This measurement of cell cycle status is related to $P\left(t\right)=2^{\int_{t_{n}}^{t}\alpha \left(s\right)ds}$, the solution of the differential equation for doubling of a population, *dP*/*dt* = *ln*(2)α(*t*)*P*. However, we utilize the measurement *c*(*t*) since, in the above mathematical system, we are considering the fate of a single cell instead of a population.

Equations 2–4 describe a hypothetical trajectory (fate) of a single cell which is genetically determined and automatically proceeds along cellular time, *g*, free of any perturbing influence, such as genetic alterations and environmental factors. Equations 2 and 3 describe the parallel process of a cell’s proliferation [α*_p_*(*t*)] and differentiation [*k_p_*(*t*)]. Equation 4 represents cellular time (*g*, generation), which is determined by factors related to cell division such as telomere length, and depends on physical time (*t*, in months, and related to patient age). Equations 5–7 incorporate the hypothetical trajectory, perturbations from it, and resistance to these perturbations as part of homeostasis. Additional explanation of the rationale of these equations were provided previously (Dai et al., 2011).

### Outline of cell properties

Each cell’s status is described by four quantities: proliferation potential (α), differentiation coefficient (*k*), resistance potential (*r*), and generation number (*g*). A cell lineage begins with the birth of an initial progenitor cell at time *t* = 0. Its physical position within the lineage is given by the number of divisions the cell is removed from the initial progenitor cell (*d*). An alternate measurement of progression is used to measure a cell’s biological progression along the differentiation pathway (*g*). This parameter may be viewed as a measurement of how a cell perceives the passage of time, which may not necessarily sync with the number of divisions its lineage has undergone. Progression of a cell’s *g* value is accompanied by the gain of additional mutations and a corresponding alteration in α*_p_* and *k_p_*, which denote behaviors inherent to position along the differentiation pathway (Table 1).

An individual cell’s proliferation potential is denoted by α(*t*), and is distinguished from its programed rate that is inherent to its position along the differentiation pathway [α*_p_*(*t*)]. The cell has a draw toward this inherent rate which is reflected by *d*α*/dt* ∝ α*_p_*(*t*) − α(*t*), but may be influenced by other environmental effects (such as hormones). The strength of this restorative force is defined by the cell’s differentiation coefficient (*k*). Cells early in the lineage have a limited ability, due to their similarity with the initial progenitor cell, to maintain homeostasis with respect to properties inherent to the differentiation pathway. Conversely, this ability is increased, consistent with their similarity to the fully differentiated cell type, for cells late in the lineage. This idealized restorative strength is denoted *k_p_* and is inherent to a cell’s position along the pathway. Mutations alter this ability, resulting in the cell’s *k*-value. The cell’s resistance potential (*r*) defines its ability to resist deviations from normal proliferative behavior, and cells early in the pathway have a weak resistance to alterations in proliferative behavior, while those later in the pathway will have a strong resistance, provided there are few strong mutations affecting the cell. A more thorough description of these terms has been provided previously (Dai et al., 2011).

### Environmental and mutational effects

Simulations are performed with β ∼*N*(5, 0.5^2^) to represent relatively low hormone level with constant mean (μ = 5) and SD = 0.5 to indicate a slight variation of hormone levels among individual cells, consistent with a typical postmenopausal hormone level. A fixed and typical mean β value allows us to focus on the role of genetic diversity (accumulation of *m_i_* in an individual cell) among the population. The importance of overexposure of estrogen, and other environmental factors in endometrial oncogenesis will be reported in separate manuscripts. We also assume two mutations per cell division in accordance with the hypothesis under consideration. As a consequence of evolution in epithelial cells due to immortalization and de-differentiation, a clinically detectable tumor is defined as a mass of at least 10^6^ cells derived from an initial progenitor cell. In this early exploration of the model, the initial progenitor cells within the uterine epithelium are assumed identical and independently follow the seven equations.

## Results

### Cellular proliferation and differentiation in the uterine epithelium

**Figure 1:**
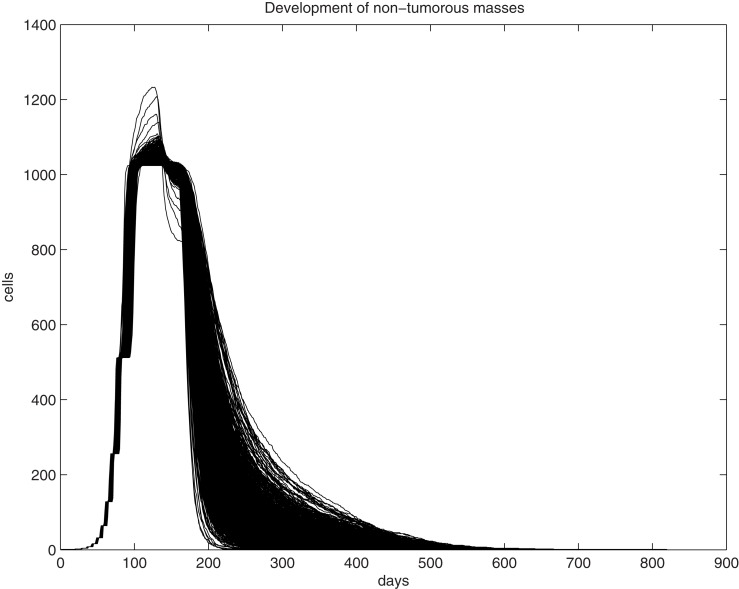
**Graphic illustration of trajectories for the number of living cells within a clone and its lifespan (days) over time**. One-thousand trajectories, each as the result of clonal expansion from a single progenitor cell, are shown.

We first examine the clonal expansion from a progenitor cell in order to understand the life cycle of epithelial cells in the uterine epithelium. Simulations are initiated with an initial progenitor cell born through asymmetric division or differentiation of a tissue stem cell. The clone is allowed to proliferate until it dies out. The size curve of each clone over time for a single progenitor cell is fairly consistent, however, as can be seen from 1,000 randomly selected trajectories generated through simulation of the fate of 10^6^ progenitor cells (Figure 1). We find that the peak size of each clone ranges from 1,024 to 1,277 cells, with a median value of 1,033.5 cells and a standard deviation of 16.2. This can be interpreted both as the typical fate of a clone spawned from each progenitor cell and is the common physiological scenario. Thus, for any cell and any clone, their lifespan is limited and they follow a predictable course and fate. One feature of tissue homeostasis, interpreted as the maintenance of a relatively stable cell number, is largely accomplished by the balance between two mechanisms, the constant commitment of tissue stem cells to produce new cells and the limited lifespan (number of generations) of individual cells to allow cell death. Thus, a significant extension of a cell’s lifespan and a substantial expansion of its descendant size beyond the typical physiological range will disrupt tissue homeostasis and serve as an early step of oncogenesis. Analysis of simulations on 10^6^ progenitor cells has shown that the lifespan of the clones was found to have a wider range, varying between 205 and 901 days, with a median of 576 days and a standard deviation of 67.5, a significant extension from the observation in Figure 1. Immortalization will be expected if the simulation involves a significantly larger population.

Indeed, a further analysis of the fates of 305,505,000 progenitor cells resulted in the detection of 8 tumors, translated into an endometrial cancer incidence of 94 tumors per 100,000 menopausal women, similar to the epidemiological data of 78 per 100,000 women based on the 2008 SEER database for all races of age ≥50. This also yielded an empirical probability of 2.61862 × 10^−8^ [95% confidence interval (1.13053 × 10^−8^, 5.15998 × 10^−8^)] that a progenitor cell will spawn a primary tumor under the experimental conditions. Our simulation has shown the progression from common physiological tissue regeneration (in 10^3^ randomly selected progenitor cells) to partial immortalization (in 10^6^ progenitor cells) and the occurrence of neoplasm (in 3 × 10^8^ progenitor cells), demonstrating oncogenesis as a seemingly rare stochastic event which occurs only in a sufficiently large number of simulations under specific environmental (hormone) conditions. More importantly, this experiment indicates that a random mutation rate of two per cell division may be sufficient for sporadic endometrial cancer.

### Phylogenetic tree analysis

**Figure 2:**
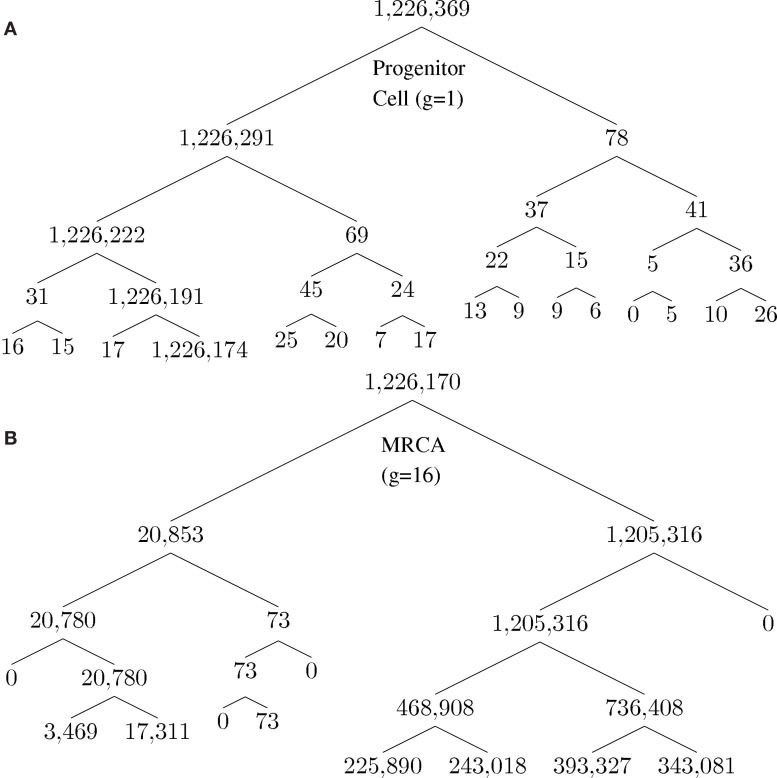
**Phylogenetic tree for illustration of lineage relationship during the earliest stage of oncogenesis**. The number indicates the size of descendants in the tumor from the cells (nodes). **(A)** The lineage map formed within the first five divisions; **(B)** a subset of the phylogenetic tree, centered on the most recent common ancestor (MRCA) of the tumor of generation 16.

A unique ID is assigned to each cell born during the lifespan of the clone. The cell passes information about its lineage to each daughter cell after division by assigning the daughter cell the ID 10*x* + *i*, where *x* is the ID of the parent and *i* is either 1 or 2, unique to each daughter. Figure 2 shows how a tumor arises from a clone. Figure 2A shows the number of descendants from each cell in the first five generations starting from a progenitor, which eventually give rise to the most recent common ancestor (MRCA) of a tumor, where division 1 denotes the birth of the progenitor cell through an asymmetric division of a stem cell. Note that there is one dominant branch with more than 10^6^ descendants, whereas other branch points have few descendants (the node with 78 descendant cells in the tumor), which coexist with the tumor and survive longer than a typical normal cell because of slow progression in the completion of senescence (and cell death) due to a low α value. Cell feature analysis shows that they have a high *k-*value (still differentiated) and are not immortalized since their generation number is less than 12 [see Cells within the tumor that are not descendant from MRCA(0.995) in Appendix]. Figure 2B shows the MRCA of the tumor at generation 16 with subsequent divisions demonstrating different lineages with varying descendant sizes. Thus, there is remarkable clonal heterogeneity in that the number of descendant cells varies substantially in different branches.

### The phenotypic heterogeneity of the tumor-initiating cancer cells

The heterogeneity of a tumor during its clonal development was analyzed by considering distributional information aggregated from 74 tumors generated through this mathematical model. The heterogeneous features in individual cells are described based on three criteria: the immortalization status by generation number *g*, proliferation status by proliferation potential α, and differentiation status by differentiation coefficient *k*. The median time required to form masses of size 10^6^ cells was found to be approximately 270 days.

We utilized a phylogenetic analysis of each tumor in order to examine the development of endometrial cancer. The MRCA of *x* × 100% of the mass of 10^6^ cells will be denoted by MRCA(*x*). We first considered the number of divisions between the MRCA(*x*) and the initial progenitor cell. The lifespan typical for a normal cell clone is commonly estimated to be between 10 and 12 divisions, where cells would reach the fully differentiated cell type and enter senescence. Some cells, as our analysis shows, remain in the process of their senescence for some time before their death. Data for MRCA(*x*) from the 74 tumors is presented in Table 3. MRCA(1) is found to be 1 division for each mass, however MRCA(0.999) and MRCA(0.995) jump to a median of 16.7 and 17.2 divisions, respectively, which indicates the immortalization (Table 3).

**Table 3:** **(A) *d* For MRCA(*x*); (B) α for MRCA(*x*); and (C) *k* for MRCA(*x*)**.

MRCA(*x*)	Median	SD	Min	Max
**(A)**				
1	1	0	1	1
0.999	16.70	2.36	9	22
0.995	17.20	2.09	11	22
0.99	17.30	2.05	11	22
0.95	18.18	2.34	13	23
0.90	18.72	1.99	14	23
0.80	19.04	2.18	14	24
0.70	19.62	2.35	14	25
0.60	20.22	2.41	14	27
0.50	21.18	2.43	15	28
**(B)**				
1	2.95	0.004	2.80	3.09
0.999	3.43	1.25	1.13	5.95
0.995	3.66	1.41	1.13	6.26
0.99	3.73	1.41	1.13	6.26
0.95	4.23	1.22	1.66	7.39
0.90	4.49	1.39	1.66	7.39
0.80	4.65	1.49	1.66	7.39
0.70	6.02	1.97	1.66	7.95
0.60	6.66	1.72	2.24	7.95
0.50	6.53	1.44	4.24	9.12
**(C)**				
1	1.90	0.015	1.65	2.24
0.999	0.23	0.05	0	1.43
0.995	0.18	0.03	0	0.90
0.99	0.17	0.03	0	0.90
0.95	0.11	0.02	0	0.76
0.90	0.08	0.01	0	0.30
0.80	0.07	0.01	0	0.30
0.70	0.07	0.01	0	0.30
0.60	0.05	0.005	0	0.30
0.50	0.03	0.002	0	0.27

The phenotype of MRCA(*x*) can be further defined by the values for its proliferation potential (α) and its differentiation coefficient (*k*) in addition to the generation number in Table 3. Data for these values are provided in Tables 3, respectively. The evolution of low *k*-values is the underlying mechanism of uncontrolled tumor growth due to loss of differentiation, as this parameter defines the differentiation status of a cell. As this value decreases, the cell becomes more susceptible to any external stimulation such as hormones. The MRCA for all cancer cells in a tumor must be a cancer cell if, as we assume based on consensus in the literature, cancer is monoclonal in origin (Weinberg, 2007; Hanahan and Weinberg, 2011). We define, based on the analysis of the formation of 74 tumors, a TICC as a cell with the median properties of MRCA(0.995). Although there is substantial heterogeneity in the phenotypes among MRCA(0.995)s, these cells are immortalized with generation number *g* between 11 and 22, proliferative with α between 1.1 and 6.3, and most important of all, de-differentiated with *k* between 0 and 0.9. We define a typical TICC as a cancer cell with the following median features: *k* = 0.18, α = 3.66, *g* = 17. Consequently, we define a typical tumor-initiating cancer stem cell (TICSC) as a TICC with completely undifferentiated status: *k* = 0, α = 3.66, *g* = 17. Using the features of a typical TICC, simulation of the fate of 10,300 TICCs showed a 71.7% probability that they will spawn a tumor, while the corresponding TICSC had roughly a 94% probability.

### Distributional analysis of the heterogeneity of a tumor formed by a TICC

The primary tumor formed from a TICC is a heterogeneous mass of cells. Continuous proliferation of cancer cells have resulted in the accumulation of an increasing number of genetic mutations and produced a cancer cell population with an enormous genetic diversity, which will drive further tumor evolution and progression. This genotypic and phenotypic variability increases the difficulty for a therapeutic intervention, such as targeted therapies aiming at a specific genetic alteration, to kill all cancer cells. A distributional analysis of a single tumor formed from a TICC was performed in order to analyze the spectrum of phenotypes and overall properties of the tumor. Table 4 describes the division number *d* of each cell within the clinically detectable mass, that is, the number of divisions that have passed between the cell and the initial progenitor cell. Note that most cells possess at least *d* = 30, with a median value of *d* = 44, indicating that almost all cancer cells in a tumor are immortalized.

**Table 4:** ***d*-Value cdf for cancer cells in a clinically detectable tumor**.

*d*_0_	28	30	32	34	36	38	40
Pr(*d* ≤ *d*_0_)	1.34E−5	8.81E−5	6.28E−4	2.89E−3	1.18E−2	4.11E−2	0.126
*d*_0_	41	42	43	44	45	46	47
Pr(*d* ≤ *d*_0_)	0.207	0.328	0.489	0.673	0.818	0.927	0.993

The intra-tumor heterogeneity is also illustrated by the distribution of *k*-values within the mass. A terminally differentiated cell will typically have a *k* ≈ 4.0, indicating a strong capability to maintain homeostasis. However, Table 5 shows that the median *k*-value within the mass is only 0.3, with no values above 1.7, illustrating the de-differentiation (malignant transformation) that the cells have undergone. Interestingly, there are approximately 7% of cancer cells with *k* = 0, indicating that they are completely undifferentiated, and are the cancer stem cell portion in the tumor (see Evolution of low *k*-values in the mass in Appendix). Finally, we consider the heterogeneity in cell proliferation through analysis of the distribution of proliferation potential among cancer cells within the mass in Table 6. We observe that 98% of cells are proliferative [α(*t*) > 0], with a median value of α = 10.3.

**Table 5:** ***k*-Value cdf for cancer cells in a clinically detectable tumor**.

*k*_0_	0	0.1	0.2	0.3	0.4	0.5	0.6	0.7	0.8
Pr(*k* ≤ *k*_0_)	0.0695	0.210	0.351	0.500	0.641	0.762	0.857	0.922	0.962
*k*_0_	0.9	1.0	1.1	1.2	1.3	1.4	1.5	1.6	1.7
Pr(*k* ≤ *k*_0_)	0.984	0.994	0.998	0.9994	0.99986	0.99996	0.999991	0.999999	1

**Table 6:** **α-Value cdf for cancer cells in a clinically detectable tumor**.

α_0_	−7.5	−5	−2.5	0	2
Pr(α ≤ α_0_)	8.33E−5	1.50E−3	6.25E−3	1.91E−2	4.26E−2
α_0_	4	5	6	7	8
Pr(α ≤ α_0_)	8.72E−2	0.120	0.163	0.216	0.280
α_0_	9	10	11	12	13
Pr(α ≤ α_0_)	0.359	0.451	0.556	0.669	0.784
α_0_	14	15	16	17	18
Pr(α ≤ α_0_)	0.883	0.954	0.989	0.9992	1

### Analysis of the median properties of tumors formed by TICCs and TICSCs

**Figure A3:**
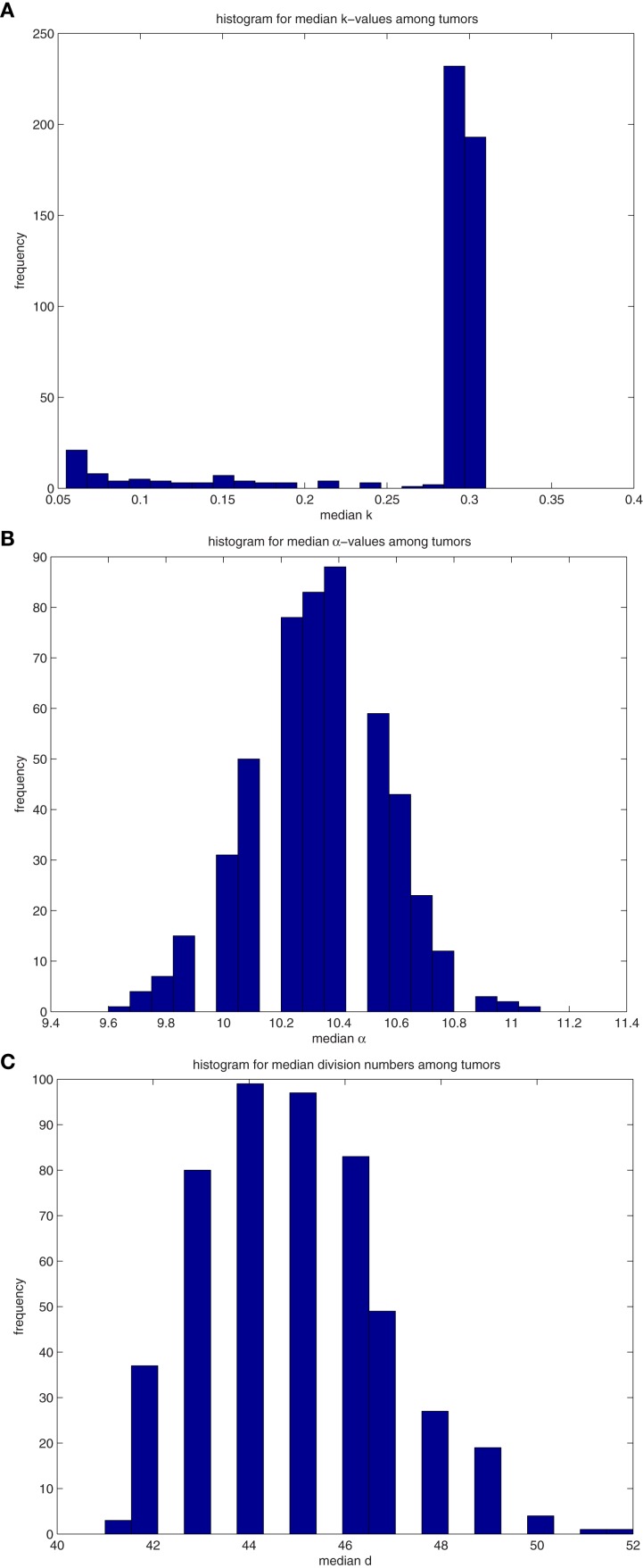
**(A)**Histograms for the median values of differentiation coefficient *k* from 500 tumors spawned from TICCs. **(B)** Histograms for the median values of proliferation potential α from 500 tumors spawned from TICCs. **(C)** Histograms for the median values of division number *d* from 500 tumors spawned from TICCs.

We extend the above analysis to 500 tumors generated from TICCs. The median properties of each tumor are recorded, and the distribution of these values is then analyzed. Table 7 lists statistical information for the median properties of the 500 tumors produced by TICCs, with corresponding histograms presented in the Figures A3(A)–(C) in Appendix. Based on this information, we define a median cancer cell (MCC) in a clinically detectable tumor as a cell with the properties: *k* = 0.295, α = 10.3, *g* = 45. The tumors appear to be very similar with respect to median proliferation potentials and division numbers, both of which have statistical properties similar to normal distributions. However, the distribution of median *k*-values deserves more attention. Whereas most tumors had median *k*-values similar to the single TICC tumor examined above (median and mean of *k* around 0.3), some of the median values are significantly lower, approaching *k* = 0. These tumors are poorly differentiated and particularly aggressive, with the capability to undergo rapid proliferation when receiving environmental stimulation conducive to growth. For the purpose of controlled comparison, we define a median cancer stem cell (MCSC) in a clinically detectable tumor as a MCC with a completely undifferentiated feature: *k* = 0, α = 10.3, *g* = 45.

**Table 7:** **Properties of the distributions of median values of cancer cells among 500 tumors derived from (A) TICC and (B) TICSC**.

Property	Median	Mean	SD	Skewness	Kurtosis
**(A)**					
*k*	0.295	0.271	0.0672	−2.34	6.87
α	10.3	10.326	0.236	7.10E−4	3.15
*d*	45	44.978	1.90	0.484	2.96
**(B)**					
*k*	0.31	0.297	0.0469	−4.13	18.72
α	10.4	10.36	0.207	0.0835	3.42
*d*	43	43.92	1.79	1.63	7.39

**Figure A4:**
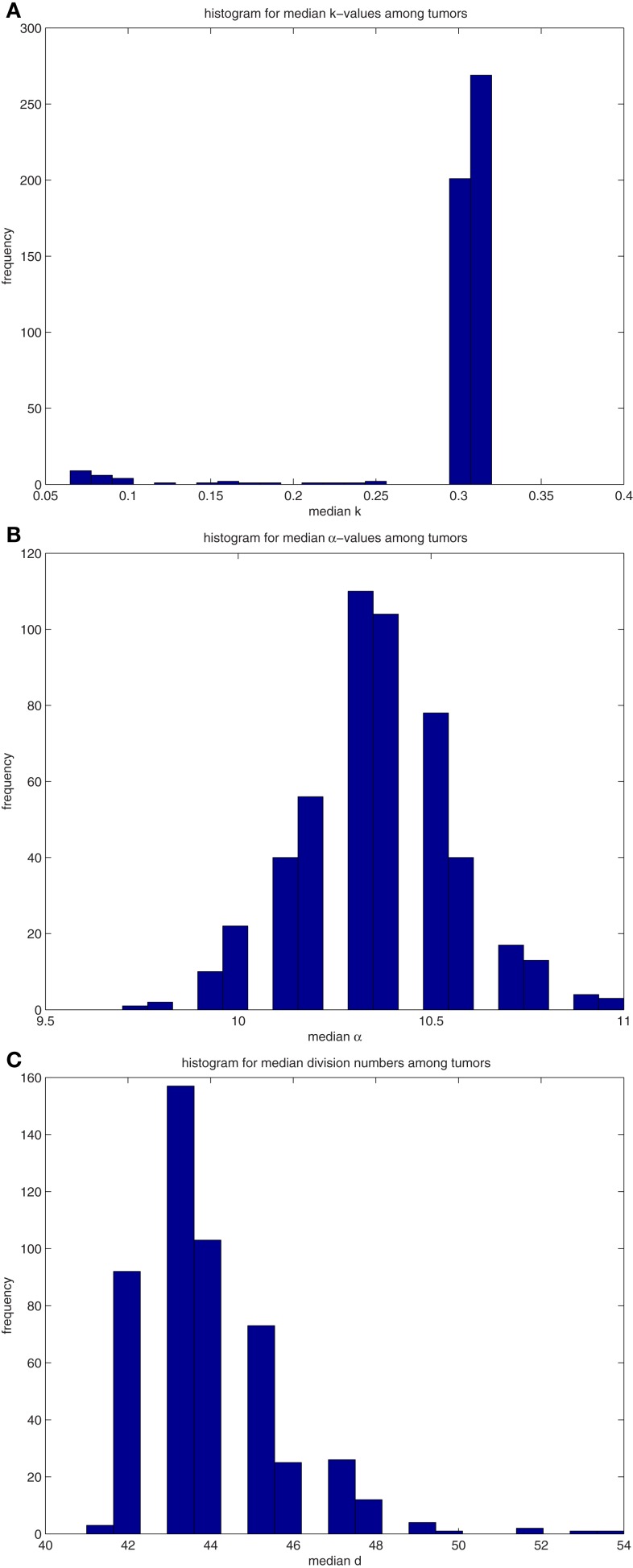
**(A)**Histograms for the median values of differentiation coefficient *k* from 500 tumors spawned from TICSCs. **(B)** Histograms for the median values of proliferation potential α from 500 tumors spawned from TICSCs. **(C)** Histograms for the median values of division number *d* from 500 tumors spawned from TICSCs.

A similar analysis was performed on 500 tumors spawned from TICSCs, with distributions for the median properties presented in Table 7 and illustrated as histograms in the Figures A4(A)–(C) in Appendix.

### Comparison of the median properties of tumors among those formed by a TICC vs. TICSC

The types of distributions derived from the median properties from the 500 tumors are unknown. However, the non-parametric two-sample Kolmogorov–Smirnov test (Hollander and Wolfe, 1999) can be utilized to examine whether the empirical distributions of a specific property are statistically equivalent among primary tumors formed from either a TICC or TICSC.

The distributions of median values of *k*, α, and *d* among tumors formed by TICCs were tested against the corresponding distributions among tumors formed from TICSCs. In each case, we find that the null hypothesis can be rejected to at least a 99% confidence (α: *p* = 0.00428, *k*: *p* = 1.3 × 10^−157^, *d*: *p* = 9.5 × 10^−17^). We conclude that a qualitative difference exists between tumors formed from a cancer cell as compared to those formed from a cancer stem cell. However, it should be noted that the median of the median properties appear to be similar for the primary tumors regardless of whether they were spawned from a TICC or TICSC.

## Discussion

Carcinogenesis as an evolutionary consequence can be viewed as the result of environmental selection among billions of genetically diverse cells in a tissue. Theoretical approaches have the unique strength of modeling the behavior of individual cells in a tissue and to construct the landscape of a dynamic and diverse cell population in order to identify and define a much smaller spectrum of cancer cells. This prospective strategy is necessary and should be complementary to the common biological approach to characterize the decisive role of a single or a few genetic alterations.

We have developed a mathematical model to simulate evolution in an epithelial tissue with an individual cell as the basic member and the entire tissue as the population. This model is unique in that it assigns quantitative value (due to varying *m_i_*) to genetic features in each individual cell and a quantitative value (α) of growth advantage translated from combined effect of genetic features $\left(\overset{n}{\underset{i=1}{\sum }}m_{i}\right)$ and environmental factors (β) in a single cell at a given time. Hormone level (β), the dominant environmental factor in uterine epithelium, is fixed at a level typical for the majority of menopausal women. The influence of these environmental factors will be further explored in a future manuscript.

Our simulations have shown that a rate of two random mutations per cell division has the potential to provide sufficient genetic diversity for enabling evolution among the simulated uterine epithelial cells. The rare event of immortalization and malignant transformation is observed when the simulation has been performed for a sufficiently large number of progenitor cells with the resultant cancer incidence comparable to the level found in epidemiological data. Our model of normal cells in the uterine epithelium gives phylogenetic context to the clonal progression of a TICC into a clinically detectable tumor and, more generally, simulates the longitudinal and prospective process of tumor development, including evolution in a normal cell population, the birth of the TICC and formation of a tumor. Cancer cells and cancer stem cells are defined based on their major features which distinguish them from normal (non-cancer) cells such as the status of de-differentiation (*k-*value), uncontrolled proliferation (α value), and immortalization (*g* value). Since all these three criteria are quantitatively expressed, a meaningful definition of cancer cells and cancer stem cells at the single cell level and of a tumor at the clinical level can be derived by their probability to form a tumor and a metastatic lesion in defined environmental conditions. The empirical and pathological terms of benign tumor, precancerous lesion, well-differentiated tumor (good outcome), and poorly differentiated cancer can be quantitatively and progressively described by the probability for tumor progression and development of metastatic diseases under a specific genetic and environmental set of conditions. Furthermore, interaction of genetic factors (*m_i_*) and environmental factors (β) can be quantitatively studied along a timeline to determine their combined effect (probability) on tumor development. Additionally, our model is built upon the description of single cells, and can thus be used to describe intra-tumor heterogeneity based upon features of individual cells. Description of cell-specific features is important to understand the heterogeneous nature of a tumor and to identify the cells with the greatest potential for metastasis. While the difference in heterogeneity between tumors can be described statistically as we did in Section “Comparison of the Median Properties of Tumors Among Those Formed by a TICC vs. TICSC,” documentation of the features of individual cells, such as immortalization, proliferation, and de-differentiation, also allows investigation of the malignant potential of individual cells, for instance, to investigate the difference in metastatic potential between a cancer stem cell and a non-stem cancer cell.

This manuscript is primarily focused on the understanding of genetic diversity in evolution. The important role of environmental factors in the selection of cells with fitness has not been presented, and remains a relevant subject for this model. Additionally, our model remains a single cell model which should be further developed to include terms to address cell–cell interactions and the role of tissue structure. For instance, angiogenesis and the molecular mechanisms underlying migration of cancer cells from the primary tumor are extremely important factors to determine cancer cell migration dynamics and the efficacy of metastasis.

Taken together, our model has provided a novel approach to demonstrate genetic diversity and evolutionary dynamics in a normal cell population at the earliest stage of oncogenesis. Cell-specific description of genotypes and phenotypes has also provided a potentially powerful tool to quantitatively analyze and understand the evolutionary process in tumor development.

## Conflict of Interest Statement

The authors declare that the research was conducted in the absence of any commercial or financial relationships that could be construed as a potential conflict of interest.

## Figures and Tables

**Figure A1:**
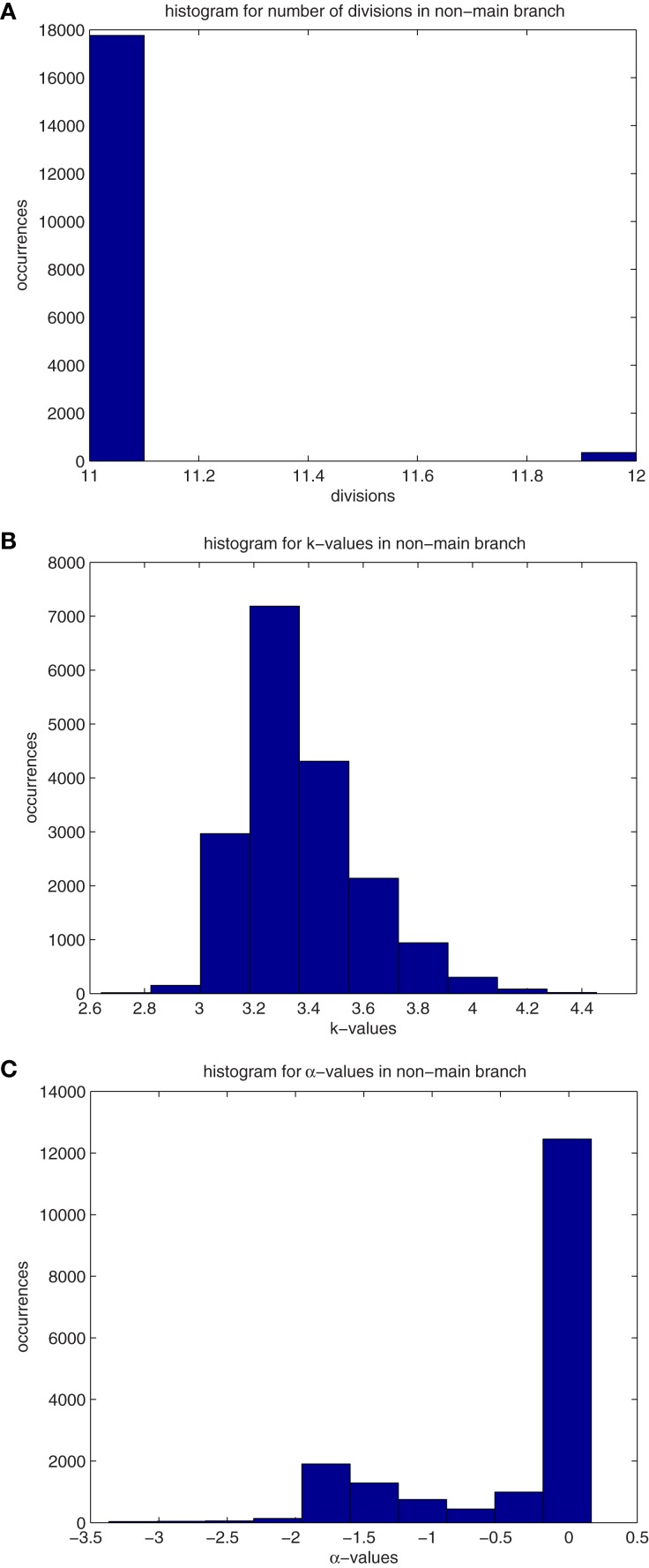
**(A)**Histograms for the median values of division number, *d*, of cells that are not descendant from the main branch. Values are recorded from 74 primary tumors. The properties in these histograms are similar to those of “normal” cells, ones that are following the differentiation pathway. **(B)** Histograms for the median values of differentiation coefficient, *k*, of cells that are not descendant from the main branch. Values are recorded from 74 primary tumors. The properties in these histograms are similar to those of “normal” cells, ones that are following the differentiation pathway. **(C)** Histograms for the median values of proliferation potential, α, of cells that are not descendant from the main branch. Values are recorded from 74 primary tumors. The properties in these histograms are similar to those of “normal” cells, ones that are following the differentiation pathway.

**Figure A2:**
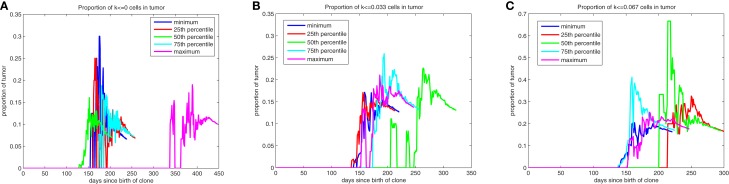
**Quantile plots for the evolution of low *k*-values during the formation of primary tumors**. Proportion of the mass with [**(A)**-left] *k* = 0, [**(B)**-middle] *k* ≤ 0.033, and [**(C)**-right] *k* ≤ 0.067.

## Appendix

### Results

#### Cells within the tumor that are not descendant from MRCA(0.995)

There are a few cells remaining in the developed tumor that are descendant from early branch points during the development of the mass (Figure A1(A) in manuscript). All other cells are from the main branch forming the tumor, with the branch point spawning the tumor found to be MRCA(0.995). Among the 74 tumors under analysis, the number of cells not emanating from the main branch was found to range from 38 to 728, with a median value of 244.824. These cells have undergone between 11 and 12 divisions, with a median of 11.0196 (Figure A1(A)). Their *k*-values are quite high (Figure A1(B)), and values range from 2.64 to 4.45, with a median of 3.37. These cells are following the inherent physiological lifespan, and are near the point of senescence. They are undergoing or preparing to undergo apoptosis. This is evidenced by their proliferation potentials (Figure A1(C)) which ranges from −3.37 to 0.164 with a median value of −0.44. These “remnant” cells are still following the normal status of development and will soon die out.

#### Evolution of low *k*-values in the mass

The evolution of low *k*-values within the mass as it develops is illustrated by quantile plots in Figure A2, where the quantiles are determined based on the proportion of the tumor with these low *k*-values at the time the mass reaches 106 cells. After these low *k*-values appear, they quickly (over a period of approximately 1 month) form subpopulations comprising roughly 25% of the mass. This evolution is likely occurring within the dominant subpopulation that is driving the formation of the tumor. We find that roughly 7% of the mass will comprise cells with *k* = 0, cells that have lost all draw toward behavior inherent to the pathway and thus have stem cell-like behavior, with approximately 13% having *k* < 0.033 and 18% having *k* < 0.067. Moreover, the dynamic nature of the composition of the tumor during its early development is illustrated in Figure A2.

**Table A1:** **Data for the max sizes of masses that eventually die out**.

Experiment	Median	Mean	SD	Skewness	Kurtosis
A	5	16496.5	83716.5	6.78706	52.5286
B	5	13981.3	75298.7	7.90778	75.1809
C	5865	123907	220817	2.04835	6.35482

**Table A2:** **Proportion of masses with max sizes surpassing thresholds**.

Experiment	10 K	50 K	100 K	200 K	300 K	400 K	500 K	600 K	700 K	800 K	900 K
A	0.092	0.053	0.042	0.024	0.02	0.015	0.013	0.008	0.005	0.001	0
B	0.08	0.05	0.038	0.023	0.014	0.01	0.009	0.005	0.003	0.002	0.002
C	0.47	0.35	0.287	0.211	0.157	0.122	0.094	0.069	0.042	0.025	0.01
